# Left ventricular free wall rupture as a result of delayed presentation of an inferior ST-elevation myocardial infarction due to fear of COVID-19: case report

**DOI:** 10.1186/s13019-021-01495-x

**Published:** 2021-04-22

**Authors:** George H. Nasr, Diana Glovaci, Andrew Mikhail, Steven Sinfield, Kevin Chen, Hardikkumar Patel, Michael Johl, Bharath Chakravarthy, Siddharth Singh, Fabio Sagebin, Ailin Barseghian El-Farra

**Affiliations:** 1 Department of Medicine, University of California, Irvine, USA; 2 Department of Medicine, Division of Cardiology, University of California, Irvine, USA; 3 Department of Emergency Medicine, University of California, Irvine, USA; 4 Department of Anesthesia & Perioperative Care, University of California, Irvine, USA; 5 Department of Surgery, Division of Cardiothoracic Surgery, University of California, Irvine, USA

**Keywords:** Left ventricular free wall rupture, Inferior STEMI, Cardiac tamponade, Pericardial effusion, MI complications, CTA, COVID-19, Case report

## Abstract

**Background:**

Left ventricular free wall rupture (LVFWR) is a rare complication after myocardial infarction and usually occurs 1 to 4 days after the infarct. Over the past decade, the overall incidence of LVFWR has decreased given the advancements in reperfusion therapies. However, during the COVID-19 pandemic, there has been a significant delay in hospital presentation of patients suffering myocardial infarctions, leading to a higher incidence of mechanical complications from myocardial infarctions such as LVFWR.

**Case presentation:**

We present a case in which a patient suffered a LVFWR as a mechanical complication from myocardial infarction due to delay in seeking care over fear of contracting COVID-19 from the medical setting. The patient had been having chest pain for a few days but refused to seek medical care due to fear of contracting COVID-19 from within the medical setting. He eventually suffered a cardiac arrest at home from a massive inferior myocardial infarction and found to be in cardiac tamponade from a left ventricular perforation. He was emergently taken to the operating room to attempt to repair the rupture but he ultimately expired on the operating table.

**Conclusions:**

The occurrence of LVFWR has been on a more significant rise over the course of the COVID-19 pandemic as patients delay seeking care over fear of contracting COVID-19 from within the medical setting. Clinicians should consider mechanical complications of MI when patients present as an out-of-hospital cardiac arrest, particularly during the COVID-19 pandemic, as delay in seeking care is often the exacerbating factor.

**Supplementary Information:**

The online version contains supplementary material available at 10.1186/s13019-021-01495-x.

## Background

Left ventricular free wall rupture (LVFWR) is a rare complication that can occur after suffering a myocardial infarction (MI). The incidence of LVFWR has decreased dramatically over the years with the increased use of reperfusion strategies such as percutaneous coronary intervention (PCI) and fibrinolytic therapy, with an overall incidence ranging from 0.8% to 6.2% [1]. LVFWR is most likely to occur 1–4 days after the initial myocardial insult, and is one of the more deadly complications of MI [2]. The early diagnosis of LVFWR is critical and point of care ultrasound (POCUS) can help establish the diagnosis quickly by revealing evidence of pericardial effusion and tamponade.

The COVID-19 pandemic has had a dramatic effect on life in the United States (U.S.) and has significantly impacted and burdened the medical community. An increasing number of patients have avoided seeking medical care due to fear of contracting COVID-19 from the medical setting. This is especially concerning for patients who may be suffering from myocardial infarctions, as a delay in treatment can lead to devastating consequences. Since the onset of the COVID-19 pandemic in March 2020 in the U.S., there has been a much higher incidence of mechanical complications, such as valvular or ventricular wall ruptures, from myocardial infarctions due to delay in treatment [3–6]. Here we present a case of a patient who suffered a left ventricular free wall rupture as a mechanical complication of myocardial infarction due to delay in seeking treatment over fear of contracting COVID-19.

## Case presentation

**Fig. 1:**
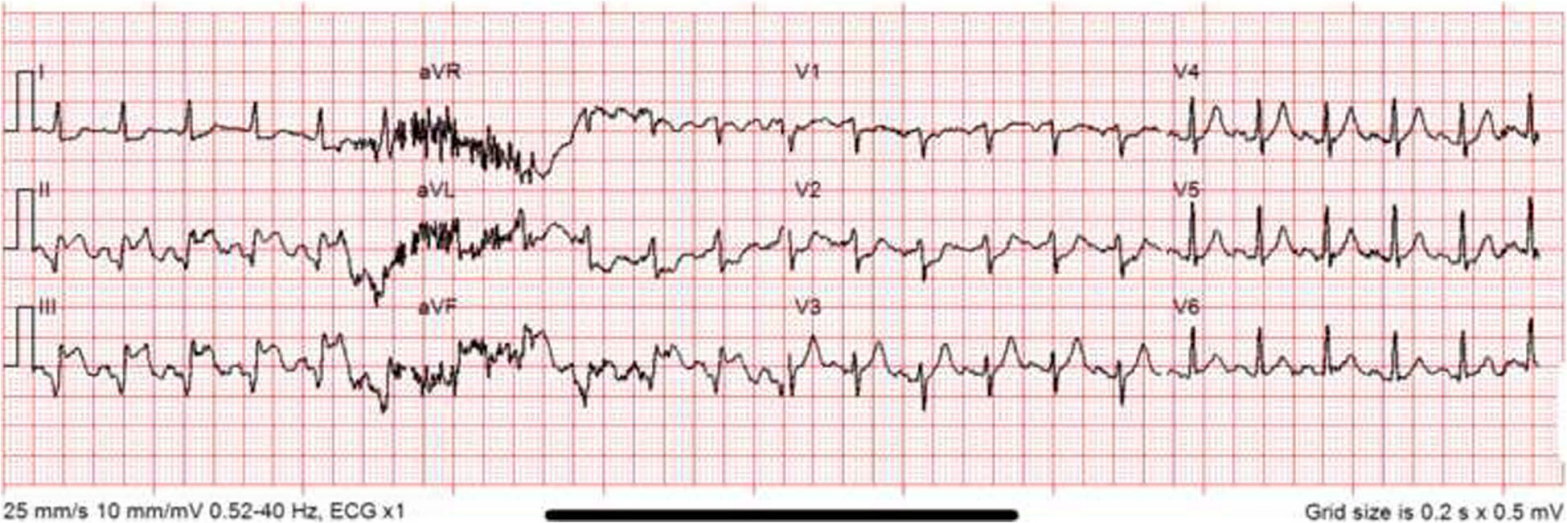
ECG by EMS performed on arrival at patient’s home showing ST-segment elevations and pathological q-waves leads II, III, and aVF with reciprocal ST-segment depressions in leads I and aVL consistent with delayed inferior myocardial infarction

**Fig. 2:**
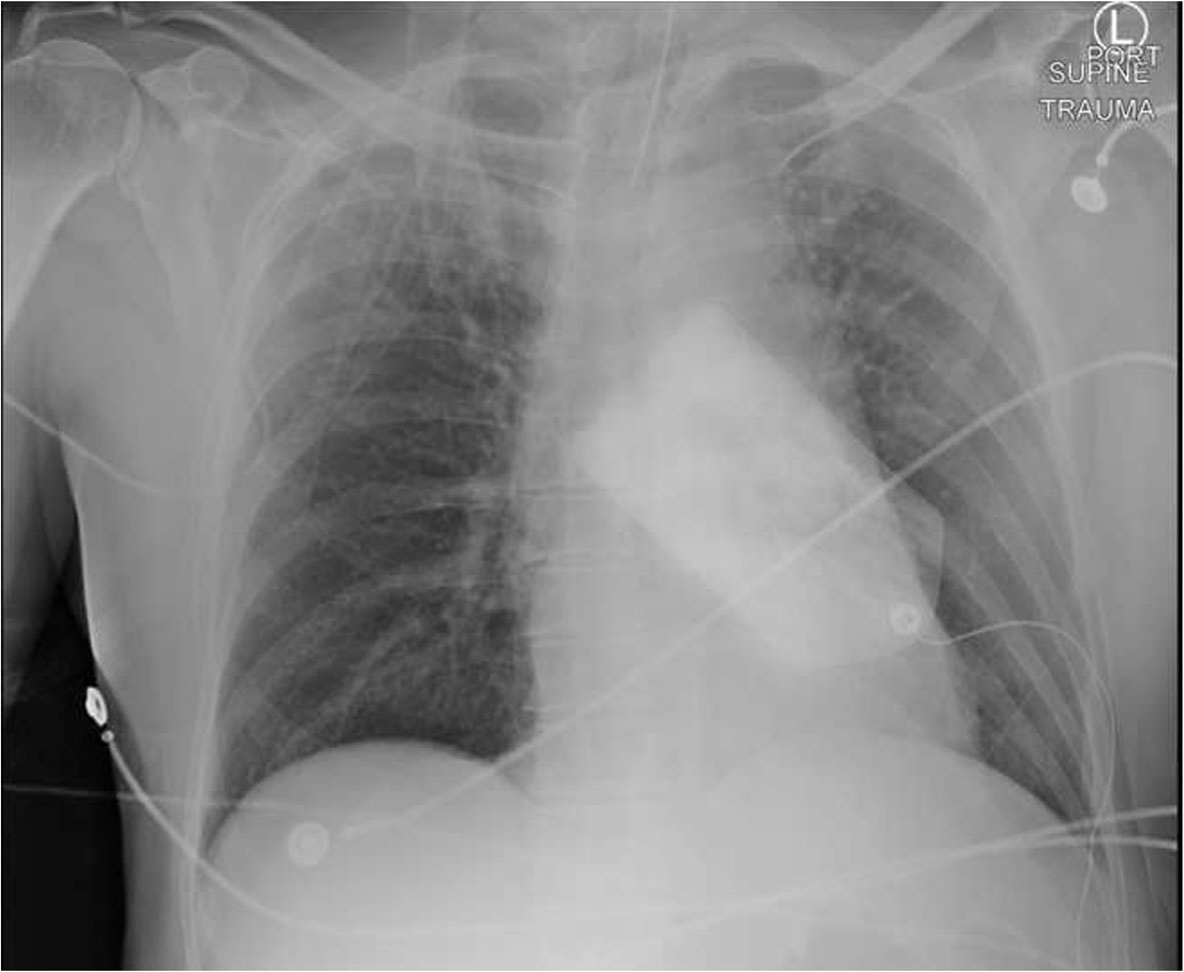
CXR on presentation revealing a widened mediastinum

**Fig. 3:**
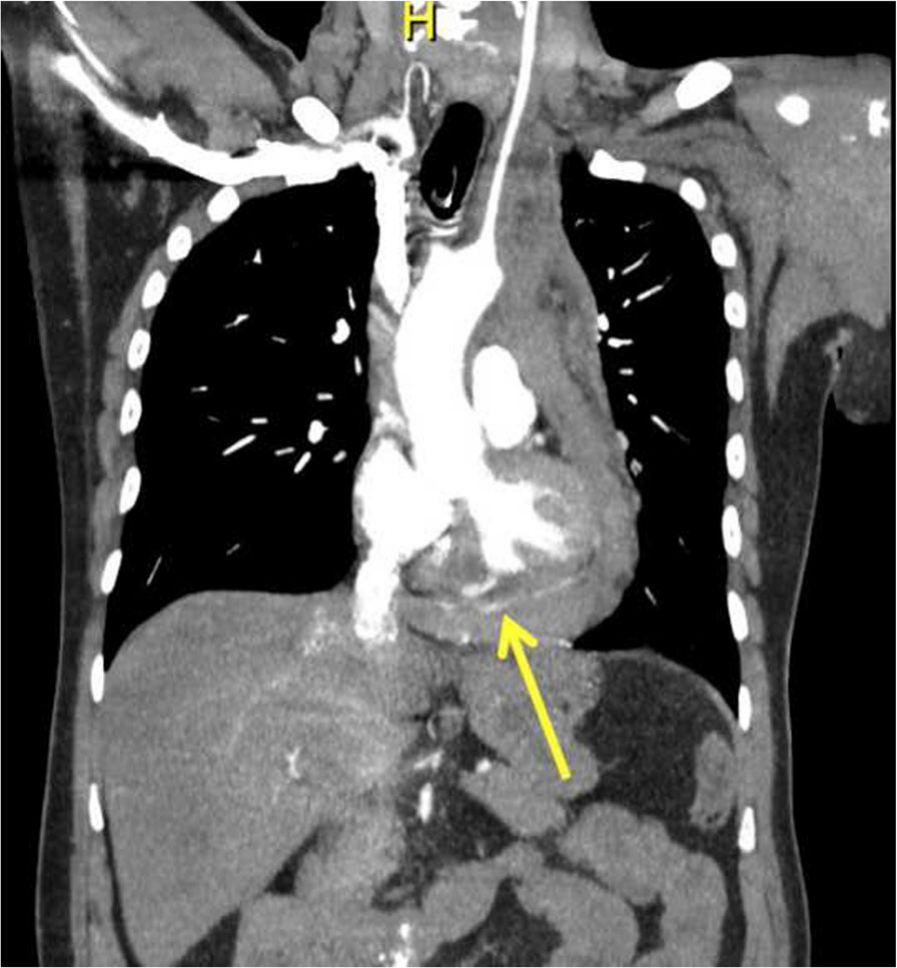
Coronal view of Aortic CTA revealing contrast leakage in the pericardium (yellow arrow) consistent with LVFWR

**Fig. 4:**
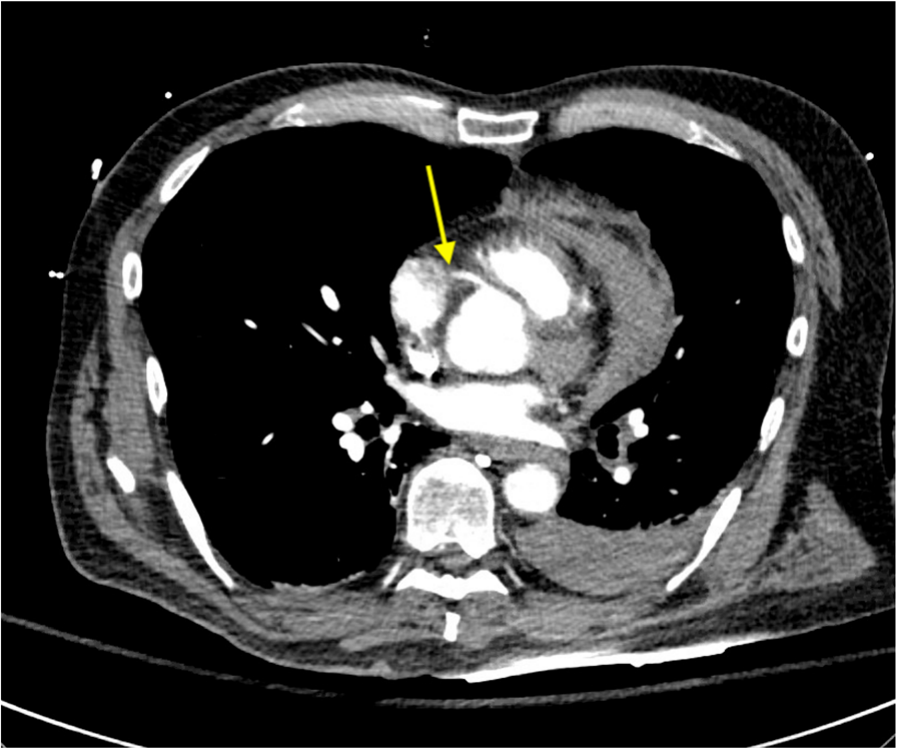
Axial view of Aortic CTA demonstrating occlusion of the proximal segment of the right coronary artery (yellow arrow)

A 67 year old male with a history of heavy smoking was witnessed by his wife to have suddenly collapsed at home. The patient had been complaining of chest pain for a few days but did not seek medical care due to fear of contracting COVID-19 from within the medical setting. When emergency medical services (EMS) arrived to his house, the patient was complaining of severe crushing chest pain and a 12 lead electrocardiogram (ECG) performed revealed ST-segment elevations and pathological q-waves in leads II, III, and aVF with reciprocal ST-segment depressions in leads I and aVL, consistent with inferior ST-segment elevation myocardial infarction (STEMI) (Fig. 1). En route to the hospital the patient subsequently lost pulses and underwent cardiopulmonary resuscitation (CPR) for 3 min prior to arrival to the emergency department (ED). On arrival to the ED, the patient underwent 10 more minutes of CPR until return of spontaneous circulation (ROSC) was achieved. After appropriate donning of personal protective equipment (PPE) by ED personnel, the patient was intubated and point of care (POC) ultrasound revealed a depressed ejection fraction (EF) and a large pericardial effusion with tamponade physiology. A pericardiocentesis was attempted twice, however aspiration of the pericardial effusion was unsuccessful which raised concern for a hematoma formation from a possible contained old rupture. A chest x-ray revealed a widened mediastinum (Fig. 2). The patient was taken emergently for computed tomography angiography (CTA) scan of his aorta. The CT scan revealed active extravasation through a small channel in the inferior wall of the left ventricle into the pericardial cavity consistent with left ventricular myocardial perforation. There was also evidence of reflux of contrast into the inferior vena cava, distal hepatic veins, and right renal vein suggestive of low cardiac output in the setting of acute cardiac tamponade, left ventricular perforation and hemopericardium (Fig. 3). The right coronary artery was noted to be opacified in the proximal segment with significant acute thrombosis (Fig. 4). The patient lost pulses multiple times in the ER and was too hemodynamically unstable to be taken to the catheterization lab for PCI at the time. In an effort to prevent further cardiac arrests, he was immediately taken to the operation room (OR) for further exploration and possible evacuation of the large pericardial effusion.

**Fig. 5:**
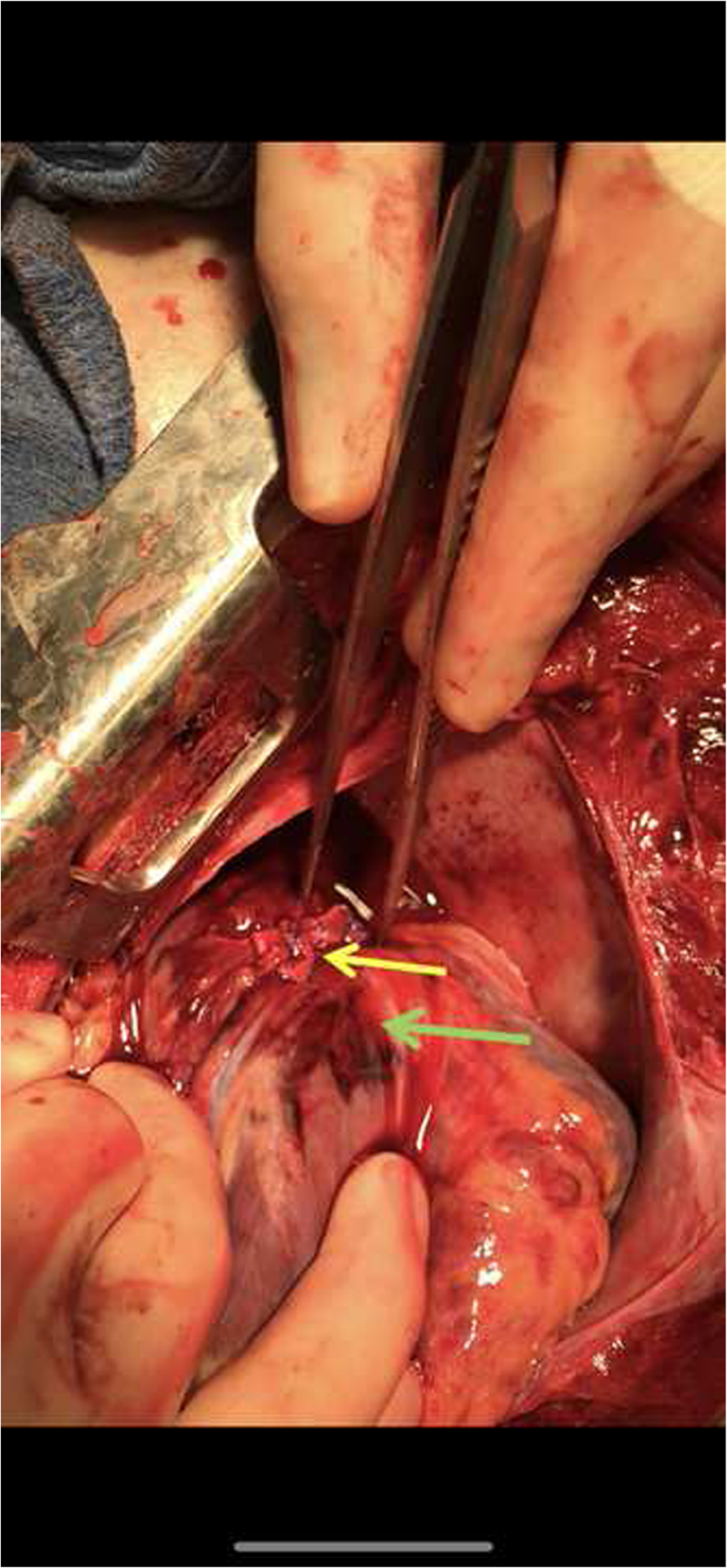
OR view of attempted suture in the open free wall of the diaphragmatic surface of the left ventricle (yellow arrow). Also, note the bruising surrounding the friable myocardial around the inferior free wall of the left ventricle (green arrow)


In the OR, the hemopericardium was evacuated through an emergent subxiphoid incision and the chest alongside the pericardium was opened. One liter of blood was evacuated. Given ongoing blood loss, a large catheter was directly placed into the right atrium for transfusion and another catheter was directly placed into the aorta to transduce blood pressure. The patient underwent aggressive resuscitation with a max systolic blood pressure of 50 mmHg, and his left ventricle was noted to be empty throughout this time. On closer examination of the left ventricle, there was a large free wall rupture and surrounding fat pad hematoma on the diaphragmatic surface of the left ventricle, likely from the inferior STEMI he suffered. They also noted similar discoloration and a hematoma lateral to the left anterior descending (LAD) artery. The open free wall of the left ventricle was attempted to be sutured to control the bleeding, however, the myocardium was noted to be extremely fragile and could not hold the suture (Fig. 5). Intraoperative transesophageal echocardiogram (TEE) following evacuation of blood revealed inferior and septal wall hypokinesis, no evidence of a flap suggestive of aortic dissection, and multiple cardiac arrests requiring CPR via intraoperative open thoracotomy cardiac massage (Video 1 A-B). After about another hour of attempted resuscitation to control the bleeding, his systolic pressure was no longer able to be generated and the patient eventually expired on the table.

**Video 1A.** TEE transgastric short axis view (left) and transgastric long axis view (right) showing hypokinetic inferior wall (yellow arrow)


**Video 1B.** TEE midesophageal four chamber view showing attempted CPR via intraoperative cardiac massage

## Discussion

The overall incidence of free wall rupture has dramatically decreased with advancements and widespread use of PCI [1]. Over half the deaths of LVFWR occur as an out-of-the-hospital sudden death [7]. The mortality rate of LVFWR is extremely high, estimated to be around 88.2% [8].

It is important to make note of mechanical complications of acute MI, such as LVFWR, when a patient presents as an out-of-hospital cardiac arrest. Although rare, LVFWR is more common than other complications such as papillary muscle or interventricular septal rupture [1]. Free wall rupture may occur sooner than anticipated and a quick bedside POC ultrasound can help identify the pericardial effusion to further guide management. Although mortality is extremely high, attempted early reperfusion and surgical management is the mainstay of treatment.

The most common presentation of a free wall rupture is an acute MI most often in the anterior and lateral region of the left ventricle with an associated pericardial effusion, usually complicated by cardiac arrest. One of the leading risk factors for LVFWR is absence of immediate perfusion [1]. During the COVID-19 pandemic, it has been demonstrated that the median time for STEMI presentation from symptom onset to hospital arrival has significantly increased from 2 h to 15 h [9]. This delay in presentation is associated with an increased incidence of post-MI mechanical complications during the COVID-19 pandemic, as seen in two large observational studies conducted in Italy and Germany [4, 6] and multiple case reports in the U.S. [3, 5]. Given that the patient had been complaining of chest pain for a few days and had pathological q-waves in the inferior leads on his ECG, it is likely that this dramatic presentation of free wall rupture was due to delay in seeking medical care.

The COVID-19 pandemic has had a major impact on the U.S. healthcare system and there have been multiple reports of its’ influence on myocardial infarction presentation. It has been shown that since the start of COVID-19 lockdowns in March 2020, there has been a significant 38% decrease in STEMI presentations in the U.S. and a 48% decrease in hospitalization rate for myocardial infarction [10, 11]. It is hypothesized that this is due in part to social distancing and patients avoiding hospital settings in fear of contracting COVID-19. It is important for clinicians to consider mechanical complications of MI, such as LVFWR, when patients present as an out-of-hospital cardiac arrest, particularly during the COVID-19 pandemic where there is often a delay of presentation from initial onset of symptoms.

## Conclusion

LVFWR, albeit rare, occurs 1–4 days after suffering an MI. The incidence of LVFWR has decreased over the past decade with advancements in reperfusion therapy, however, there has been an increased incidence of LVFWR during the COVID-19 pandemic as patients delay seeking care over fear of contracting COVID-19 from within the medical setting. Clinicians should consider mechanical complications of MI when patients present as an out-of-hospital cardiac arrest, particularly during the COVID-19 pandemic.

## Data Availability

Data sharing is not applicable to this case report as no datasets were generated or analyzed in our article.

## Abbreviations

LVFWR: Left ventricular free wall rupture
MI: Myocardial infarction
STEMI: ST-segment elevation myocardial infarction
PCI: Percutaneous coronary intervention
POCUS: Point of care ultrasound
US: United States
ED: Emergency department
EMS: Emergency medical services
ECG: Electrocardiogram
CPR: Cardiopulmonary resuscitation
ROSC: Return of spontaneous circulation
EF: Ejection fraction
CTA: Computed tomography angiography
OR: Operation room
LAD: Left anterior descending
TEE: Transesophageal echocardiogram
